# Neuropathologies of the self and the right hemisphere: a window into productive personal pathologies

**DOI:** 10.3389/fnhum.2013.00472

**Published:** 2013-08-20

**Authors:** Todd E. Feinberg

**Affiliations:** Departments of Psychiatry and Neurology, Albert Einstein College of Medicine, Yeshiva UniversityNew York, NY, USA

**Keywords:** neuropathologies of the self, ego boundaries, right hemisphere, Capgras syndrome, somatoparaphrenia

The *neuropathologies of the self* (*NPS*; Figure 1) is a proposed broad grouping of various syndromes in which the common factors are that a demonstrable focal brain lesion(s) or dementia causes an alteration in the patient's personal identity or personal relationships between the self and the world. The NPS may include many conditions (some of which are highlighted in the Figure 1) but some of the better known are the *delusional misidentification syndromes* (*DMS; Capgras and Frégoli syndromes, DMS for the mirror image); somatoparaphrenia; and phantom boarder syndrome* (Feinberg, 2001, 2009a,b, 2010, 2011a; Feinberg et al., 1999; Feinberg and Keenan, 2005).

**Figure 1:**
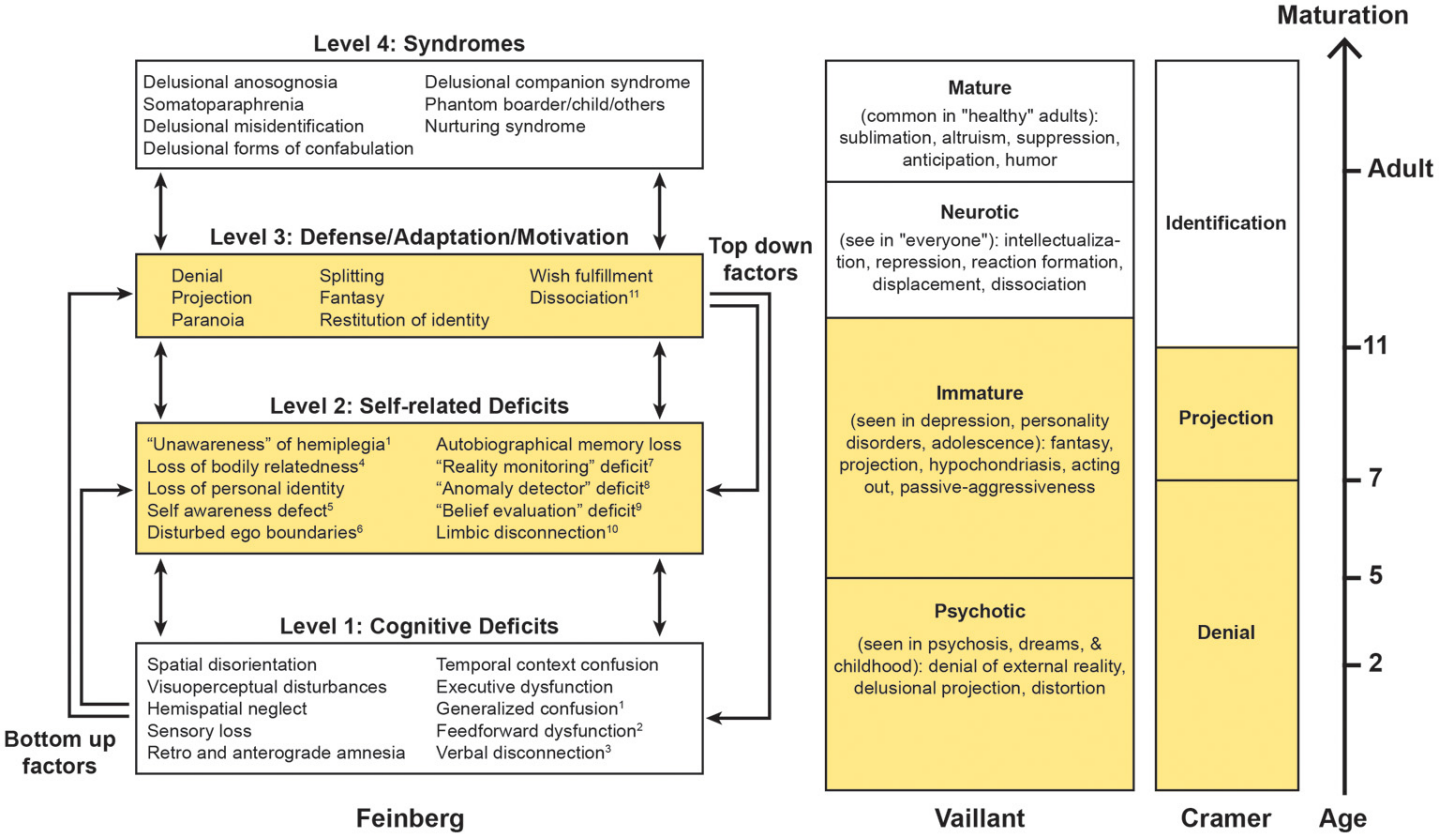
**Based upon Feinberg (2010, 2011a); Vaillant (1977, 1992, 1993) and Cramer (1991, 2006).** On the left is a hierarchical four-tiered model of representative factors contributing to some of the neuropathologies of the self. Some cognitive (level 1) deficits are specifically linked to certain syndromes, while self-related deficits and positive features may be applied to many syndromes. A temporal dimension emerges as syndromes evolve from the interaction of multiple lower level negative and higher level positive factors. This model is compared with the hierarchical model of the defenses proposed by Vaillant (1977, 1992, 1993) and the developmental model of Cramer (1991, 2006). Areas in yellow represent the corresponding immature psychological functions across models. ^1^Levine (1990), Levine et al. (1991); ^2^Heilman (1991), Heilman et al. (1998). ^3^With reference to anosognosia and asomatognosia see Geschwind (1965), Gazzaniga (2000); ^4^For the neural representation of body ownership see Feinberg et al. (1990), Tsakiris (2009), Vallar and Ronchi (2009); ^5^Stuss (1991); ^6^ Feinberg et al. (2005), Baier and Karnath (2008), Feinberg (2009a,b), Feinberg et al. (2010); ^7^Johnson (1991); ^8^Ramachandran (1995); ^9^Davies et al. (2005), McKay et al. (2005), Coltheart (2007); ^10^With reference to Capgras syndrome see Alexander et al. (1979), Ellis and Young (1990), Ellis et al. (1997), Ellis (1998); ^11^With reference to DMS see Christodoulou (1977); Christodoulou (1986).

## A hierarchical model of the NPS

The NPS differ from purely cognitive disorders in that the mistaken beliefs in NPS are more delusional, personally idiosyncratic or bizarre, more influenced by the patient's motivations and personal relationships, and less tied to a specific domain of neurological impairment (multi-modal). Although the misidentifications, delusions, and confabulations in the NPS entail many (Jackson, 1884) *negative factors—*that is defects cause by the *absence* of particular neurological functions—the NPS also have numerous *positive*, productive, defensive, and motivational features based upon what the brain can and does do. In order to take into account both the negative and positive aspects of the NPS, I proposed a model (Figure 1) based upon a network of negative and positive factors and bottom–up and top–down interactions within a hierarchy of cognitive deficits and other psychological functions (Feinberg, 2010, 2011a).

On the hierarchically lowest rung (level 1) are the cognitive deficits—depending upon the particular syndrome—that play a role in the creation of various disorders. For example, in patients with somatoparaphrenia, who most commonly display delusions and confabulations about a paralyzed side of the body or arm “believed or experienced as absent” (Gerstmann, 1942; Vallar and Ronchi, 2009), frequent negative contributing factors to the syndrome include sensory loss and hemispatial neglect (Vallar and Ronchi, 2009; Feinberg et al., 2010) that could contribute to a loss of feeling of relatedness to the limb. In a similar fashion, in other DMS, executive dysfunction, memory disorders, and visuoperceptual and visuospatial deficits have been implicated as important, lower level, precursor, or contributing factors (for references, see Figure 1).

At the next hierarchical (level 2) are some of the specifically self-related deficits that are important in the etiology of the NPS. Like level 1 factors, these are negative factors, but at this level they are specifically linked to self-related functions. For example, in the development of somatoparaphrenia, deficits in self-awareness of limb actions (Baier and Karnath, 2008) and spatial representation of the body (Vallar and Ronchi, 2009) have been hypothesized to play a role. More generally, other level 2 negative factors that could contribute are a failure of self-monitoring as part of the frontal dysexecutive syndrome (Stuss and Benson, 1985; Stuss, 1991; Stuss et al., 2001, 2005), a “reality monitoring defect” (Johnson, 1991; Johnson et al., 2000) an “anomaly detector” defect (Ramachandran, 1995) or a deficit in a hypothetical “belief evaluation system” (Coltheart, 2005, 2007; Davies et al., 2005; McKay et al., 2005).

We have suggested (Feinberg and Keenan, 2005; Feinberg et al., 2005; Feinberg, 2009a,b, 2010, 2011a) that a critical negative feature at this level is an alteration in the *permeability or regulation of the self-boundaries*. This can be an *under-relatedness* to personally significant aspects of the self as occurs in Capgras syndrome and somatoparaphrenia, or an *over-relatedness* to selected aspects of the world where the patient inappropriately over-incorporates neutral aspects of the environment into the self as occurs in the Frégoli syndrome.

While levels 1 and 2 are comprised of *negative factors* that are based upon cognitive and functional *impairments*—functions that the brain is not doing or cannot do—on level 3 we find productive, motivational, and defensive *positive factors*—that is processes that the brain is doing and can do, often in *response* to levels 1 and 2 deficits that often serve as precursors. On this level psychological defenses—processes that are based upon psychodynamic and psychoanalytic theories (Vaillant, 1977, 1992, 1993)—are particularly important.

Psychological defenses can be organized according their degree of psychological maturity and their typical age of appearance (Figure 1). In the hierarchical model of the psychological defenses proposed by Vaillant (1977); Vaillant (1992); Vaillant (1993), *denial, delusional projection, paranoia*, and *distortion* are the hierarchically lowest, most primitive and most pathological defenses. These are followed by *projection* and *fantasy* that are considered “immature defenses” because they make their appearance later in child development (Figure 1, column 2).

Along similar lines, Cramer (1991, 2006) proposes a hierarchy of defenses along a developmental time line. In her view, psychological defenses occur as a necessary and adaptive part of child development (Figure 1, column 3). Cramer finds that beginning roughly around age 3 the earliest defense to develop is *psychological denial* and this remains the major defense until about age 7, at which point *psychological projection* (where the individual deals with unacceptable emotions or thoughts by attributing them to others) becomes the dominant defense. By about age 7 denial and projection are approximately equal and after that point *identification* takes on an increasingly important role (Cramer, 1991, 2006).

Also during the developmentally earlier periods, *fantasy*—another productive feature—serves defensive and motivational functions. Just as Vaillant considers fantasy an “immature defense,” Cramer (1991, 2006) points out that *denial through fantasy* enables the child to cope with unpleasant realities and Taylor (1999) argues that the fantasy of *imaginary companions* in children, a fantasy that has many features in common with some of the delusions in the DMS, serves a variety of adaptive functions for the child such as coping with emotional trauma and anxiety.

The immature defenses and functions are the ones most relevant to the coping strategies and defenses of adults with neurological injury and NPS (Feinberg, 2009a, 2010). In order to test this hypothesis, I collected a representative series of published reports and personal cases of neurological patients with *imaginary others* (*N* = 21) and determined the presence or absence of psychological defense(s) and other potential adaptive mechanisms in each case (Feinberg, 2010; for details and narratives of these cases, see Feinberg, 2009a). In this series there was clear predominance of immature mechanisms: paranoia and wish-fulfilling fantasy were the most common, each appearing in 9 (43%) of cases, denial was detected in 5 cases (30%), and projection appeared in 2. Splitting, another primitive defense that has relevance to the creation of imaginary companions in children and is also associated with the adult neuropathology cases (Berson, 1983) appeared in 2 cases and in only 2 cases were none of these mechanisms apparent or reported.

## The NPS, the right hemisphere, and the early development of psychological defense

Why would brain injury or dysfunction activate primitive defenses? Several authors have hypothesized that increasing cognitive skills based on brain maturation leads to the progression from immature to mature defenses (Laughlin, 1970; Lichtenberg and Slap, 1972; Elkind, 1976; Chandler et al., 1978; Wallerstein, 1985; Cramer, 1991, 2006). I have argued that the disturbance in self-boundaries in the NPS creates a de-differentiation between inner and outer reality and the margins of the self and that the immature defenses are most likely to reflect this disturbance.

The neuropathological findings associated with the NPS suggest a possible mechanism for how this might occur. It has been frequently reported that many of the NPS are associated with frontal pathology especially involving the right hemisphere (Alexander et al., 1979; Feinberg and Shapiro, 1989; Förstl et al., 1991; Malloy et al., 1992; Fleminger and Burns, 1993; Burgess et al., 1996; Spangenberg et al., 1998). For instance, in one fairly large series, Burgess et al. (1996) reviewed 41 reported cases of DMS, confabulation, and reduplicative phenomena and found the highest percentage of cases had right frontal hemisphere (44%) or bilateral frontal (39%) lesions compared with only 9.7% who had left frontal lesions. Feinberg et al. (2005; see also Feinberg and Keenan, 2005) analyzed cases of DMS or delusional reduplication and found all twenty-nine observations (100%) suffered right hemisphere damage, while only 15 (51.72%) suffered from left hemisphere damage, and in 28 out of 29 of the observations (96.6%), right frontal damage was present.

In another investigation (Feinberg et al., 2010) we compared cases (all with right hemisphere lesions) with *simple asomatognosia* that showed unelaborated errors regarding the ownership of the limb, to cases with *somatoparaphrenia* that showed more extensive delusions, misidentifications, and confabulations regarding the limb (the latter features closely associated with the other NPS) and controls with neither of these syndromes. All patients with simple asomatognosia or somatoparaphrenia, as well as controls, had significant right temporoparietal involvement; however, patients with somatoparaphrenia had the overall largest lesions and significantly more right frontal involvement when compared to patients with simple asomatognosia. Further, while patients with simple asomatognosia and somatoparaphrenia had more medial frontal damage when compared to control groups, somatoparaphrenia patients also demonstrated significant right orbitofrontal damage that indicated a further role for right orbitofrontal damage in this group.

The frontal (Stuss, 1991; Stuss et al., 2001, 2005) and medial frontal (Northoff and Bermpohl, 2004; Northoff et al., 2006; Feinberg, 2009a; Feinberg et al., 2010) regions have been shown to have a significant role in several self-related functions. Along with orbitofrontal cortex, these regions are heteromodal association cortices and part of the *integrative self-system* that helps integrate the *interoself system* with the external environment (see Feinberg, 2009a, 2011a,b). This region is intimately concerned with the sense of an integrated self and the differentiation between the self and world.

In this context it is of interest that the insula, a component of the interoself system, has also been implicated in the etiology of somatoparaphrenia (Cereda et al., 2002; Baier and Karnath, 2008).

These findings further suggest that the *right* frontal regions in particular play a special role in these self-related functions. The intactness of the self-boundaries and the “ego”—defined by Vaillant (1993, p. 3) as “the adaptive and executive aspects of the human brain: the ability of the mind to integrate, master, and make sense of inner and outer reality”—play an important role in the promotion of the mature defenses (Vaillant, 1977, 1992, 1993). The prominent emergence of the immature defenses and fantasies after right frontal damage suggest a particular role for these regions in these “ego” (Freud, 1930) functions.

It is also logical that given that the primitive defenses are largely based upon verbal (productive) mechanisms and what people say about themselves and others, *and* that the delusions and defenses in the NPS that emerge after right frontal damage *are also* largely verbally expressed, it is possible that the immature defenses could be lateralized to the dominant hemisphere. Further, one could speculate that the neural structures that the mature defenses depend upon may be lateralized to the non-dominant hemisphere resulting in a parallel lateralization of the mature defenses to the non-dominant hemisphere. Alternatively, the right hemisphere may play some additional critical role in regulating or suppressing the immature defenses. Along these lines, Salas and Turnbull (2010) suggested that the emergence of immature defenses in these conditions could be caused by a failure in the regulation of arousal and negative emotional states (an “arousal regulation capacity”) which is a component of the mature defenses. If the capacity were lateralized to the right hemisphere, damage to the right hemisphere would impair this regulation.

Finally, an additional question is how in the course of neural development this occurs. Based in part upon Cramer's developmental timeline (Figure 1) I have hypothesized that in the normal course of brain maturation there may be a developmental shift from immature defensive functions and fantasies toward mature defenses and the inhibition of fantasy that critically depends upon maturational process within the right hemisphere. Once this “left brain to right brain defensive shift” occurs, the immature defenses and the use of fantasy are inhibited and the mature adult defenses are more likely to dominate.
